# Analysis of GPI-Anchored Receptor Distribution and Dynamics in Live Cells by Tag-Mediated Enzymatic Labeling and FRET

**DOI:** 10.3390/mps3020033

**Published:** 2020-04-27

**Authors:** Maria N. Balatskaya, Alexandra I. Baglay, Yury P. Rubtsov, George V. Sharonov

**Affiliations:** 1 Faculty of Medicine, Lomonosov Moscow State University, Lomonosovskiy av. 27-1, 119192 Moscow, Russia; baglay.alexandra@yandex.ru (A.I.B.); yrubtsov@gmail.com (Y.P.R.); sharonov@gmail.com (G.V.S.); 2 Shemyakin–Ovchinnikov Institute of Bioorganic Chemistry RAS, str. Miklukho-Maklaya 16/10, 117997 Moscow, Russia; 3 Institute of Translational Medicine, Pirogov Russian National Research Medical University, Ostrovitianov str. 1, 117997 Moscow, Russia; 4 Laboratory of Genomics of Antitumor Adaptive Immunity, Privolzhsky Research Medical University, 10/1 Minin & Pozharsky sq., 603005 Nizhny Novgorod, Russia

**Keywords:** FRET, membrane proteins, GPI-anchored receptors, protein labeling, fluorescent labeling, S6 tag, clustering, T-cadherin, ephrin-A1

## Abstract

The analysis of glycosylphosphatidylinositol (GPI)-anchored receptor distribution and dynamics in live cells is challenging, because their clusters exhibit subdiffraction-limited sizes and are highly dynamic. However, the cellular response depends on the GPI-anchored receptor clusters’ distribution and dynamics. Here, we compare three approaches to GPI-anchored receptor labeling (with antibodies, fluorescent proteins, and enzymatically modified small peptide tags) and use several variants of Förster resonance energy transfer (FRET) detection by confocal microscopy and flow cytometry in order to obtain insight into the distribution and the ligand-induced dynamics of GPI-anchored receptors. We found that the enzyme-mediated site-specific fluorescence labeling of T-cadherin modified with a short peptide tag (12 residues in length) have several advantages over labeling by fluorescent proteins or antibodies, including (i) the minimized distortion of the protein’s properties, (ii) the possibility to use a cell-impermeable fluorescent substrate that allows for selective labeling of surface-exposed proteins in live cells, and (iii) superior control of the donor to acceptor molar ratio. We successfully detected the FRET of GPI-anchored receptors, T-cadherin, and ephrin-A1, without ligands, and showed in real time that adiponectin induces stable T-cadherin cluster formation. In this paper (which is complementary to our recent research (Balatskaya et al., 2019)), we present the practical aspects of labeling and the heteroFRET measurements of GPI-anchored receptors to study their dynamics on a plasma membrane in live cells.

## 1. Introduction

Current knowledge of membrane receptor organization and dynamics in live cells remains far from complete, but it is important for understanding their functional activity. This is, in part, due to the resolution limit of 200 nm for conventional optical microscopy, which allows for us to study live cells. More sophisticated and precise methods, such as super-resolution and cryo-electron microscopy and X-ray crystallography, which resolve smaller structures, are challenging or unsuitable for the study of live cells.

It has been shown that the activity of several receptors depends on the oligomeric state in the membrane. For example, the dimerization of some tyrosine kinase receptors is required but not always sufficient for signal transduction [1]. There are glycosylphosphatidylinositol (GPI)-anchored receptors (more than 130 GPI-anchored proteins (GPI-APs)), according to the UNIPROT database on humans), which, without transmembrane and cytoplasmic domains, can transduce intercellular signals. The mechanisms of signal transduction by GPI-anchored receptors and the correlation between receptor activity and their structures in a membrane-associated form deserve thorough investigation. Multiple studies have demonstrated that portions of GPI-APs persist in a dimeric or oligomeric form in a steady state [2,3]. In some cases, ligand binding to GPI-APs stabilizes and increases the density of oligomeric GPI-APs followed by lipid raft dependent receptor immobilization and signaling [3,4]. In another study, artificial GPI-APs did not reside in ordered domains in the live cell plasma membrane [5]. Moreover, the distribution of the GPI-anchored receptor depends on both the GPI anchor’s composition and the structure of the ectodomain [3,6]. The detection of GPI-AP clusters in live cells in real time is a demanding task, because they exhibit subdiffraction-limited sizes and are highly dynamic [7].

A study of GPI-AP clustering in the membranes of live cells can be conducted using fluorescence-based approaches that are based on the Förster resonance energy transfer (FRET) effect between single color or multicolor labeled molecules [8]. The energy transfer efficiency E is dependent on the inverse sixth power of the distance r between the donor and acceptor, which is why FRET is a “spectroscopic ruler” [9]. Studying GPI-APs in live cells is challenging, because adequate labeling and FRET measurement protocols are not readily available. Mayor’s group showed that homoFRET (FRET between two identical fluorophores) could be more sensitive than heteroFRET (FRET between different fluorophores) for studying a small fraction (20%–40%) of clustered GPI-APs (two to four proteins per cluster) of mostly randomly distributed molecules [2]. In the homoFRET approach, all of the molecules contribute to FRET, while, in the heteroFRET setup, roughly half of the molecules in the homodimer have no FRET pair. Moreover, with heteroFRET, it is almost impossible to derive the cluster size, which is possible with homoFRET by analyzing the photobleaching curves. The authors demonstrated that the heteroFRET of the GPI-APs (donor dequenching upon acceptor photobleaching) signal is comparable to the background fluctuations of the FRET signals [2]. However, heteroFRET is compatible with conventional equipment for detecting and analyzing the fluorescence of both the donor and acceptor in two different channels. Detailed information regarding the different types of FRET measurements can be found in several reviews [10,11,12,13,14].

In our research, we compared three approaches for labeling the GPI-anchored receptors, T-cadherin and ephrin A1, and used several methods for FRET detection to obtain information regarding the distribution and ligand-induced dynamics of these receptors. The methods for the fluorescent labeling of GPI-APs should be carefully chosen to avoid the undesired effect of labeling the structure and function of the studied proteins. In this paper, we provide details of the GPI-AP enzyme-mediated fluorescence labeling, measure their interactions by FRET, and discuss the advantages that make the S6-tag based enzymatic labeling of GPI-AP a superior choice when compared to antibodies or fluorescent protein (FP) chimeras.

The FRET approaches could be used for insights into the change in the dynamics of the clustering of GPI-AP, which leads to physiological responses. In our work, we were able to observe the real-time clustering of T-cadherin after the addition of its ligand adiponectin. It was previously known that T-cadherin binds to the hexameric and high molecular weight form of adiponectin and causes beneficial effects on the cardiovascular system [15,16,17,18,19]. However, the molecular mechanisms of triggering these physiological effects have not been studied. We hypothesized that such a large ligand (25 nm) could cluster T-cadherin for inducing intracellular effects and tested this hypothesis in our work.

## 2. Materials and Methods

### 2.1. Generation of Plasmids

First, we inserted monomeric teal FP 1 (mTFP1 or TFP) as a donor and yellow fluorescent protein (tagYFP or YFP) as an acceptor near the T-cadherin GPI-attachment site [20], the same site used in some other research on GPI-APs [21,22]. Briefly, sequences that encoded the fluorescent proteins YFP or TFP, with the addition of linkers on each side and without stop codons, were inserted between the DNA sequences that coded the fifth cadherin motif and GPI-anchoring signal in human full-length T-cadherin cDNA: Leu675-His-Gly-FP-Glu-His-Ala-Arg676. We used the following primers with extra restriction sites and sequence coding linker amino acids for conventional cloning, as described earlier [20]:

R-NcoI-T-cad 5’-GATCTCCATGGAGATCTGTGATATTCGTCATGG-3’;
F-SphI-T-cad 5’-GCATGTGCATGCCAGGGTACAAGTGTGCTC-3’;
F-NcoI-YFP 5’-CTGTCACCATGGAGTTAGCAAAGGCGAGGAG-3’;
R-SphI-YFP 5’-GTATATGCATGCTCGCGGTACAGCTCGTCCAT-3’;
F-NcoI-mTFP1 5’-CTGTCACCATGGAGTGAGCAAGGGCGAGGAG-3’;
R-HinfI-mTFP1 5’-TCCATGGAGGAGGACTCCTTCAG-3’;
F-HinfI-mTFP1 5’-GAGGAGGACTCCTTCATCTACGA-3’;
R-SphI-mTFP1 5’-GTATATGCATGCTCCTTGTACAGCTCGTCCATGCC-3’.

Zhou and colleagues identified the amino acid sequences of short peptides—12 amino acid residues in length—that can serve as specific substrates for Sfp and AcpS phosphopantetheinyl transferases [23]. There are several short peptides that can be used for labeling [23,24]. We used the so-called S6 peptide and Sfp synthase (phosphopantetheinyl transferase from *Bacillus subtilis)*. Short peptide tag S6 was identified from a phage-display peptide library as one of the most efficient substrates for site-specific protein labeling catalyzed by Sfp phosphopantetheinyl transferase [23]. Therefore, we decided to use this combination of tags and enzymes in our work. We cloned the corresponding DNA sequence into the cDNAs of these proteins to create an S6-tagged T-cadherin and ephrin A1 for FRET measurement.

The first step involved the choice of insertion site and the generation of recombinant DNA constructs (the main principles discussed in Section 3.1). We built 3D models based on the primary sequences of the GPI-APs (T-cadherin and ephrin-A1) using the Phyre2 service (Protein Homology/analogY Recognition Engine 2; http://www.sbg.bio.ic.ac.uk/phyre2). Details of the algorithm are described in [25]. Visualization was performed using the Chimera software [26].

For T-cadherin, the insertion point was chosen between the T-cadherin cleavable prodomain and the first domain, which takes part in dimerization [27]. In addition, there is one amino acid linker that flanks the peptide tag. Ser139-G-GDSLSWLLRLLN-S-Ile140 (full, immature protein) was the complete amino acid sequence of the insert, which corresponds to the nucleotide sequence 5′-GGTGGCGATAGCTTAAGCTGGCTGCTGCGTCTGTTGAACAGC-3′. Cloning procedures were performed by the Evrogen company (Moscow, Russia). In brief, synthetic complementary oligos were annealed to form duplexes, which were ligated to a suitable shuttle vector (containing the CMV promoter; pIRESneo1 was obtained from Dr. K.A. Rubina, Lomonosov Moscow State University, Moscow, Russia) digested with suitable restriction enzymes (performed by Evrogen). Sanger sequencing verified the sequences of the resulting clones, and the plasmid construct was named pIRESneo1-T-cad-S6. We used pIRESneo1-T-cad-S6-S6 coded in the same site as T-cadherin for the positive control for FRET, which contains S6-peptides and two tandem copies of S6. Accordingly, the following peptide sequence was inserted into T-cad: G-GDSLSWLLRLLN-SGGGGSG-GDSLSWLLRLLN-S.

Human ephrin-A1 cDNA was cloned from MCF-7 cells, as described earlier [28]. The insertion of the S6-tag was designed to replace a stretch of five non-conservative amino acid residues (positions 60–64, ADAAM in immature ephrin-A1) that do not seem to be involved in binding with the Ephrin receptor [29]. Moreover, this flexible loop region is exposed on the surface (sticks out) of the receptor-ligand complex. The sequence of the insert is Val59-GDSLSWLLRLLN-GluE65, which is coded by the nucleotide sequence 5′-GGCGATAGCTTAAGCTGGCTGCTGCGTCTGTTGAAC-3′. The corresponding oligo duplex was ligated to the pVAX1 vector that was digested with suitable restriction enzymes to obtain the pVAX1-ephrin-S6 construct (also performed by Evrogen).

### 2.2. Transformation of Competent E. Coli Cells and the Isolation of Plasmid DNA

The protocol described in [30] was used to transform competent E. coli cells with plasmid DNA.

The isolation of plasmid DNA was carried out while using the GeneJet Plasmid Miniprep Kit (Thermo Scientific), according to the manufacturer’s protocol.

### 2.3. Transfection of Eukaryotic Cells and Selection of Clones

Human embryonic kidney cells (HEK293; the cell line was obtained from Dr. K.A. Rubina) were cultured in Dulbecco’s Modified Eagle’s Medium (DMEM, HyClone or Paneco) with an antibiotic-antimycotic solution (HyClone) and 10% fetal bovine serum (FBS, HyClone) and then incubated at 37 °C with 5% CO_2_.

The HEK293 cells were transfected by the corresponding plasmids using Lipofectamine 2000 (Invitrogen, CA, USA) to express chimeric T-cadherin or ephrin A1 tagged with S6 peptide. Transfection was done according to the manufacturer’s protocol.

The transfected cells were grown in the presence of antibiotic G418 to generate stable lines expressing tag-modified GPI-anchored receptors (400 µg/mL, Gibco). The cells with stable T-cadherin expression were obtained by the selection of cells that were stained with specific anti-T-cadherin antibodies or based on specific fluorescence of TFP/YFP by photoactivated cell sorting (FACSAria III, BD Biosciences) as described earlier [20]. The final selection efficiency of the cells expressing ephrin-S6 and T-cadherin-S6 was estimated by an FACS analysis of the cells by staining with allophycocyanin (APC)-conjugated antibodies against T-cadherin (R&D, AF3264) or primary antibodies against ephrin A1 (Santa Cruz, SC-911), followed by staining with secondary antibodies. The use of antibodies is valid in our case because HEK293 cells do not express endogenous T-cadherin and ephrin A1. We generated cell lines with the stable overexpression of S6-modified T-cadherin and ephrin A1 while using this approach.

### 2.4. Comparison of the Molecular Weight, Subcellular Localization, and Functional Activity of the Recombinant Protein

First, we compared the stability and electrophoretic mobility (molecular weight) of T-cadherin and ephrin A1 with the addition of S6-tags. Wild type untransfected cells were used as the specificity controls.

A radioimmunoprecipitation assay (RIPA) buffer (50 mM Tris-HCl pH 8.0, 120 mM NaCl, 1% NP-40, 0.5%, sodium deoxycholate, and 0.1% SDS) with protease inhibitors (Pierce) was used for cell lysis. The lysates were centrifuged at 12,000× *g* for 15 min. at 4 °C. The protein concentration was determined while using a BCA Protein Assay (Pierce). The samples were heated in a Laemmli buffer with β-mercaptoethanol at 99 °C for 5 min., loaded on gel, and then subjected to sodium dodecyl sulfate polyacrylamide gel electrophoresis (SDS-PAGE) in a 7.5% gel according to the Laemmli protocol. The protein extracts separated in gel were transferred to a polyvinylidene fluoride (PVDF) membrane (GE Healthcare) in a transfer buffer (1.92 M Tris/glycine buffer, 10% SDS, and 20% ethanol) while using a Bio-Rad transfer apparatus. The membrane was incubated in 5% nonfat dry milk in PBS (Sigma) containing 0.1% Tween-20 (Pierce) overnight at 4 °C to block nonspecific binding. Subsequently, the membrane was then incubated for 1 h with primary antibodies, washed thrice in PBST, and then incubated for 1 h with horseradish-peroxidase-conjugated secondary antibodies (IMTEK). After washing, the membrane was incubated with a chemiluminescent substrate (ECL Pico or Dura, Pierce) for one minute. Luminescence was detected with an X-ray film (Kodak) or a ChemiDoc imager (Bio-Rad). The Quantity One or Image Lab software (Bio-Rad) were used for image analysis.

The intracellular localization and surface expression of T-cadherin and ephrin-A1 were checked by antibody staining against T-cadherin or ephrin-A1, followed by confocal fluorescence microscopy and flow cytometry analyses. The functionality of the recombinant T-cadherin was confirmed by its ability to mediate calcium signaling upon binding with LDL (more details in [20]).

### 2.5. Enzymatic Labeling of GPI-Anchored Proteins

**Figure 1:**
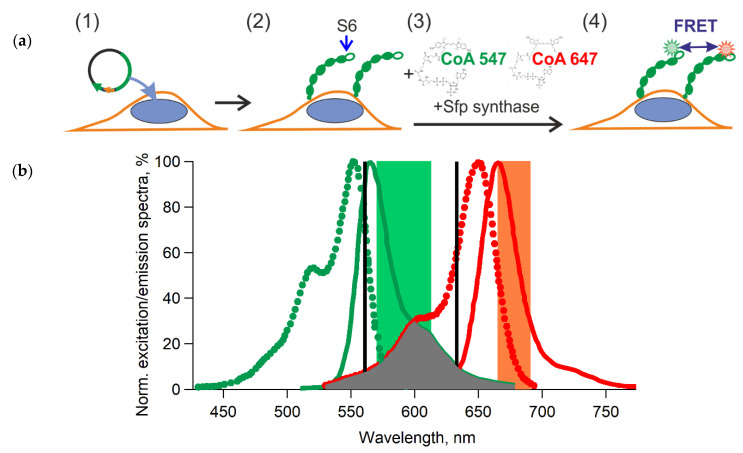
(**a**) Scheme of the fluorescent labeling of peptide tagged T-cadherin on the plasma membrane for Förster resonance energy transfer (FRET) experiments. (1) Plasmid encoding T-cadherin with the S6 peptide was introduced into the cells; (2) T-cadherin-S6 was expressed on the surface of the transfected cells; (3) covalent attachment of the fluorescent-labeled substrates CoA547 and CoA647 to T-cadherin by Sfp synthase; (4) detection of FRET between two different receptors on the membrane. (**b**) The excitation (dotted lines) and emission (solid lines) spectra of CoA547 (green) and CoA647 (red) according to the manufacturer (NEB). The overlapping integral of the donor and acceptor spectra is indicated by the grey area. The black vertical lines indicate the laser lines, and the green and orange areas indicate the detection channels used in microscopic experiments.

**Figure 2:**
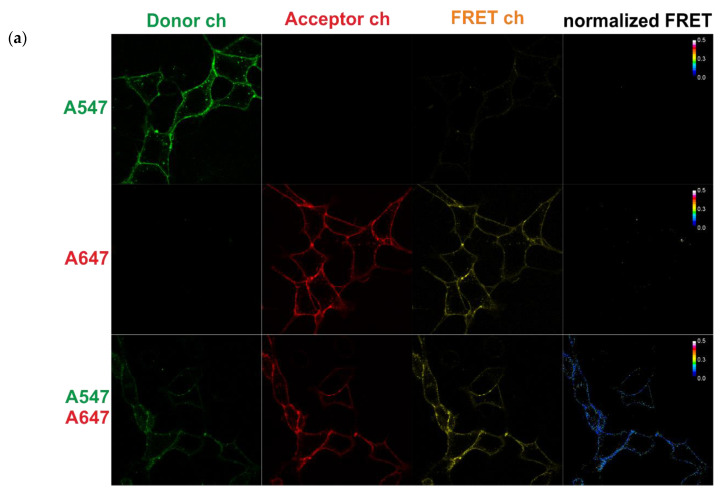

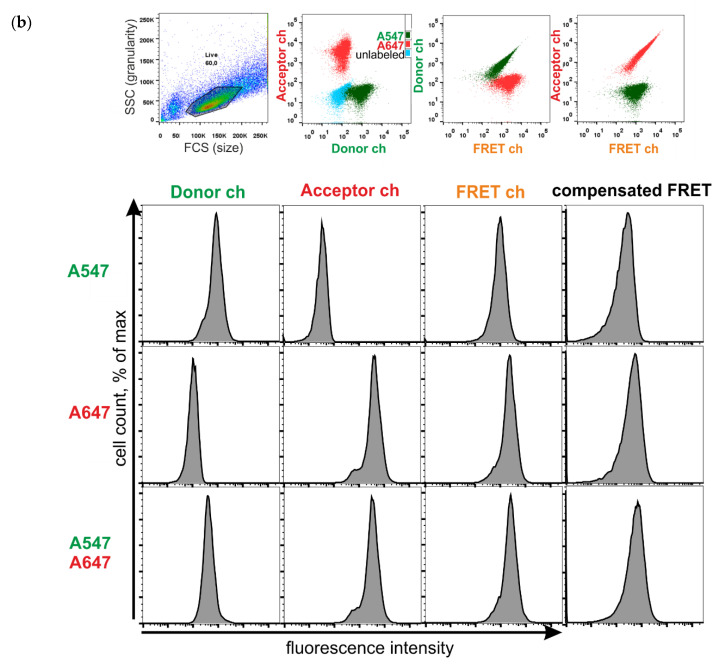
FRET-measurements of the labeled T-cadherin by confocal microscopy and flow cytometry in live cells. Live cells expressing T-cad-S6 were labeled with A547 (donor dye, green) and/or A647 (acceptor, red). (**a**) Fluorescence in the three channels: donor (green), acceptor (red), and FRET (orange). Confocal images were taken and analyzed for FRET using the Zen software. The normalized FRET signal is shown on the right. (**b**) Top—the representative plots of raw flow cytometry data. The gate has been applied to identify live cells on SSC vs. FCS density plot and use in all next plots with an unlabeled sample (cyan), a donor only sample (green, A547) and an acceptor only sample (red pixels, A647) and the histograms. The histograms represent the fluorescence of the labeled cells. Compensated FRET values were obtained using the FlowJo software.

The tagged proteins were labeled on the surface of living cells while using an enzymatic reaction (Figure 1a). For the microscopy experiments, labeling was done with the cells adhering to cover glass, while we used cell suspension for flow cytometry. The reaction conditions were the following: 1 µM Sfp synthase (NEB), substrates 2.5–5 µM CoA 547 (NEB), and/or 2.5–5 µM CoA 647 (NEB) in a DPBS solution with 0.5% BSA that was supplemented with 10 mM MgCl_2_ at room temperature (to prevent endocytosis when necessary) for 40 min. (see the step-by-step protocol in [31,32] or https://international.neb.com/). In some cases, we conducted labeling with FBS at 37 ℃ when it did not affect the outcome of the experiment. The substrates and enzyme solutions can be prepared according to the protocol in [31]. The labeled cells were washed three times and placed in DPBS with 0.5% BSA for analysis by confocal microscopy or flow cytometry. Our data show the precise membrane labeling and colocalization of labeled molecules with the membrane marker (Figure 2a; [20]).

We used two variants of CoA that were bound to fluorescent dyes to introduce different labels to the T-cadherin or ephrin-A1 molecules for heteroFRET measurements (Figure 1). We chose DY-547 (Dyomics, designated as A547) as the donor and DY-647P1 (Dyomics, designated as A647) as the acceptor. Both have high extinction coefficients in the green and red parts of the visible light spectrum, which makes it possible to avoid the use of UV and blue light for excitation and minimize the damaging effects on the cells. Moreover, it is possible to use common sources of excitation with a maximum of 561 nm for the donor (A547) and 633 nm or 640 nm for the acceptor (A647). We calculated the overlap integral of the donor and acceptor spectra *J* [cm^6^/mol] and the Förster radii *R*_0_ [cm] according to the following formulas [33]:

(1) $$J=\int^{\;}f_{D}\left(\lambda \right)\varepsilon_{A}\left(\lambda \right)\lambda^{4}d\lambda ,$$

(2) $$R_{0}=\sqrt[{6}]{8.79\times 10^{-25}\frac{\kappa^{2}\varphi_{D}}{n^{4}}J},$$

where $f_{D}$ is the donor emission spectrum normalized to an area of 1, $\varepsilon_{A}\left(\lambda \right)$ [M^−1^ cm^−1^] is the molar extinction coefficient of the acceptor at λ [cm], $\kappa^{2}$ is the dipole orientation factor, $\varphi_{D}$ is the fluorescence donor quantum yield in the absence of the acceptor, and *n* is the refractive index of the medium.

In our case, we estimated *R*_0_ while using theoretical and experimental data: $\varepsilon_{A}$ was 250,000 M^−1^ cm^−1^ at maximum (according to the manufacturer Dyomics, Jena, Germany), the overlapping integral of the donor and acceptor spectra was about 3.81·10^−13^ cm^6^/mol (according to Figure 1b and using the IgorPro software), $\kappa^{2}$ was 2/3 (according to dynamic random averaging of the donor and acceptor [33]), $\varphi_{D}$ was about 0.1 (according to Dyomics), and the *n* of DPBS with 0.5% BSA was 1.335 (according to our measurements with a refractometer (KOMZ)). Thus, R_0_ was approximately 4.4 nm. The R_0_ of this FRET pair is comparable with the typical extracellular cadherin domain length (4.5 nm) [34] and similar to the R0 values of popular FRET pairs [11].

The chosen fluorophores form a FRET pair and thus the dimerization or clustering of pairs would generate a FRET signal upon interaction between the T-cadherin/ephrin-A1 molecules containing different labels, which should give rise to FRET between these labels. The attachment of equimolar amounts of CoA derivatives with donor and acceptor dyes will result in equal amounts of donor- and acceptor-labeled receptors that are optimal for FRET measurements given the equally probable engagement of either substrate in the enzymatic reaction. 

There are many ways to measure FRET [10,11,12,13,14]. Here, we use methods based on confocal microscopy and flow cytometry.

### 2.6. FRET Measurements Using Confocal Microscopy

FRET measurements and analyses were performed using a laser scanning confocal microscope LSM 780 (Zeiss) and Zen 2010 (Zeiss). The measurements were carried out using a Plan-Apochromat 63× lens with a numerical aperture of 1.40.

One or two days before the FRET analysis, we defined the goal of the experiment (steady state receptors in a full culture media or the dynamics of receptors after adding ligands in media without FBS). We used a dish with a glass bottom (Lab-Tek Nunc, ibidi, Greiner bio-one) covered with a thin layer of type I collagen (BD or Gibco) to increase cell adhesion to study the cells by confocal imaging.

#### 2.6.1. Acceptor Photobleaching

Acceptor bleaching is often used to calculate the FRET efficiency. Selective photobleaching of the acceptor causes an increase in donor emission if FRET between the donor and acceptor occurs before bleaching. Before measurement, we selected parts of the membrane to be analyzed by defining the bleached Region of Interest (ROI) and background ROI. The fluorescence intensity was detected in an ROI of a 561-nm-excited cell membrane in the donor A547 (569–630 nm) channel for calculations and the acceptor channel (656–691 nm) to control the bleaching. After the fourth scan (baseline), we photobleached the acceptor for 30–60 s while using a 633 nm laser. Subsequently, fluorescence was detected in the donor channel, and the background fluorescence was subtracted. The FRET efficacy in the selected ROI was calculated using the following formula:

(3) $$E=\frac{I_{D}-I_{DA}}{I_{D}}\;100\%,$$

where *I_D_* is the donor fluorescence intensity after acceptor photobleaching and *I_DA_* is the donor fluorescence intensity before acceptor photobleaching. 

#### 2.6.2. Sensitized Acceptor Emission (Confocal Microscopy)

For time-lapse measurements in live cells, the photobleaching approach in unsuitable, and the sensitized emission of the acceptor can instead be used. In this case, the donor fluorophore was excited by 561 nm, the signal was collected in the donor and acceptor channels, when the acceptor was excited by 633 or 640 nm, and the signal was collected in the acceptor channel. It is possible to use a confocal microscope and a flow cytometer (see below).

Unlike acceptor photobleaching, sensitized emissions do not allow for us to simply obtain absolute FRET efficiency. Numerous FRET efficiency and index methods can be found in the literature (see, for example, the quantitative comparison in [35]). It is necessary to check crosstalk (also termed bleed-through or spillover) in excitation (excitation of the acceptor with the wavelength used for donor excitation and vice versa) and in emissions (the contribution of donor fluorescence in the acceptor detection channel and vice versa). The real signal in each channel is a linear combination of different signals. We usually use several controls to obtain the correct FRET signal via microscope or cytometer:

1. the control of autofluorescence: blank cells with their proteins of interest (POIs) tagged with S6 peptide without dyes;
2. the control of nonspecific binding dyes (the quality of washing step): cells expressing POIs without a tag with the donor + acceptor dyes;
3. the control to calculate donor spectral bleed-through: cells expressing POIs tagged with the S6 peptide with donor dye only; and,
4. the control to calculate acceptor spectral bleed-through: cells expressing POIs tagged with the S6 peptide with acceptor dye only.

For confocal microscopy, we used multi-track imaging in three channels (Figure 2a).

Track 1: The donor channel (excitation of 561 nm and emission detection of 570–613 nm) and the FRET channel (excitation of 561 nm and emission detection of 665–691 nm); 

Track 2: The acceptor channel (excitation of 633 nm and emission detection of 665–691) using the same settings and conditions. 

It is necessary to optimize the excitation laser lines, laser power, wavelength of detection, scan speed, etc. The donor excitation wavelength should not (or minimally) directly excite the acceptor. In some cases, it is better to use a shorter wavelength laser. The FRET/acceptor channel should collect a signal from the acceptor and a minimal signal from the donor. In some cases, it is better to use a longer wavelength, but spectral bleed-through into the FRET channel cannot be completely eliminated.

Microscopy images (135 × 135 µm and 512 × 512 pixels) were processed by a FRET macro in the Zen2010 software. Past researches have considered issues that affect the detection of sensitized emissions by conventional confocal microscopy and has provided corresponding open source tools that can be used for this purpose [12,36,37]. We added a few steps of averaging using the manufacturer’s procedure to obtain more reliable results (“Quick FRET Guide ZEN 2010”). We defined the ROIs (three or more) of the object and the background from each control image and recorded the obtained threshold parameters for the donor, acceptor, and FRET channels in the table. Subsequently, we used three or more control images for the donor and acceptor and obtained the average thresholds for the donor/acceptor images. We determined the maximum of the obtained average thresholds values in each of three channels and defined one set of thresholds for all of the images. The average of the threshold values was less than 13 in an eight-bit image. After background correction, we calculated the coefficients to remove spectral bleed-through between channels. We used three or more control images for the donor and acceptor to average the coefficients and then saved these parameters for further FRET analyses of the double stained specimens. All of the coefficients were independently determined for each experiment. When we only used the donor sample, we determined the ratio intensities for the FRET to donor channels (0.04–0.08) and those for the acceptor to FRET channels (0–0.005). When we used the acceptor only sample, we determined the ratio intensities for the FRET to acceptor channels (0.4–0.6), the donor to acceptor channels (0.02–0.07), and the donor to FRET channels (0.03–0.12).

In this work, we employed the normalized FRET (NFRET, Figure 2a) calculation, which corrects spectral bleed-through between channels (based on the coefficients mentioned above) and donor and acceptor concentration according to [38]. This process yielded a smaller variation in our data when compared to other methods for FRET efficiency calculations (FRETN according to [39]) and better matched the FRET kinetics measured by flow cytometry (see below). We used whole images as a single data point to calculate the average normalized FRET value for a certain experimental condition. Thanks to the selective labeling of surface receptors, we had no need to select the ROIs of the membranes in the sample images (with the donor and acceptor) and used the calculated index NFRET [38] from the entire image (512 × 512, more than 6000 non-zero pixels).

### 2.7. FRET Measurements Using Flow Cytometry

For the flow cytometry analysis, the cells were grown in culture dishes, with more than 30 thousand cells per experimental point. The cells were harvested while using a Versene solution (Paneco) for one minute, washed, and resuspended in DPBS + 0.5% BSA. Then, the cells were split into equal parts and labeled as described above. After washing, the cells were analyzed in a suspension by flow cytometry (Fortessa, BD Bioscience). We used the following excitation and detection filters to measure the fluorescence of the cells labeled with A547 and A647:

Donor ch ex 561 em 585/15
FRET ch ex 561 em 670/30
Acceptor ch ex 640 em 670/30

It is possible to use cells with either transient or stable expressions. In the first experiments, we used transiently transfected cells and prepared the following for controls: cells with donors only, cells with acceptors only, unlabeled cells, and cells with T-cadherin containing two S6 inserts that were labeled by the donor and acceptor mixture. The flow cytometry data were processed with the FlowJo 7.5 software (Treestar). In all of the experiments, we selected cells based on a forward (FSC) versus side scatter (SSC) gating plot (Figure 2b). In an experiment with transiently transfected cells, we used a gating strategy based on the control sample. All of the results showed in the figures of Section 3 based on stable line cells experiments.

As with the microscopy, we used single stained control samples to compensate for all spectral bleed-through in the other experiments. Compensation is often used in multi-color flow cytometry, and the relevant details are often discussed in the literature [40]. It is possible to apply manual or automatic compensation while using the compensation matrix via software. In our case, we did not need to correct the acceptor signal to donor channel or the donor signal to acceptor channel, but we had to remove the the donor/acceptor’s signals to FRET channel (approximately 90% and 45% in the compensation matrix, respectively) to receive the comparable median for the control samples in the FRET channel (Figure 2b).

We used the algorithm described in [41,42] to calculate FRET efficiency from the flow cytometry data. For this, we obtained median fluorescence intensity in three channels of cell population of blank sample (for the background correction) and donor/acceptor only samples. Using spectra of our donor and acceptor, we calculated FRET efficiency for T-cadherin/ephrin-A1 labeled donor and acceptor. FRET efficiency with cell-by-cell correction for autofluorescence can be calculated using software ReFlex [43].

### 2.8. Kinetics of FRET (Confocal Microscopy and Flow Cytometry)

Actually, the kinetic measurement was carried out as before. Using a confocal microscope, we measured fluorescence in the control and experimental samples in the donor, FRET, and acceptor channels and obtained NFRET. We calculated the difference between NFRET(t) and the fitted baseline NFRET. We measured fluorescence in the control and experimental samples in the donor, FRET, and acceptor channels and obtained compensated signal in FRET channel to detect FRET by flow cytometry. Our results demonstrate that all of the methods and calculations produced consistent FRET values reliably distinguishable from the controls.

The cells with T-cadherin-S6 were deprived of serum overnight, labeled, and then the baseline signals were acquired to study ligand-induced dimerization/clusterization. After 40–90 s, we added 10 μg/mL recombinant high-molecular-weight-rich human adiponectin (BioVendor, #RD172023100-C). The obtained kinetics were fitted in the IgorPro using exponential curves in the form y0 + A exp (x invTau), from which we calculated the characteristic time of the exponents as the inverse of the invTau value.

## 3. Results and Discussion

### 3.1. Different Fluorescent Labeling of GPI-Anchored T-Cadherin

**Figure 3:**
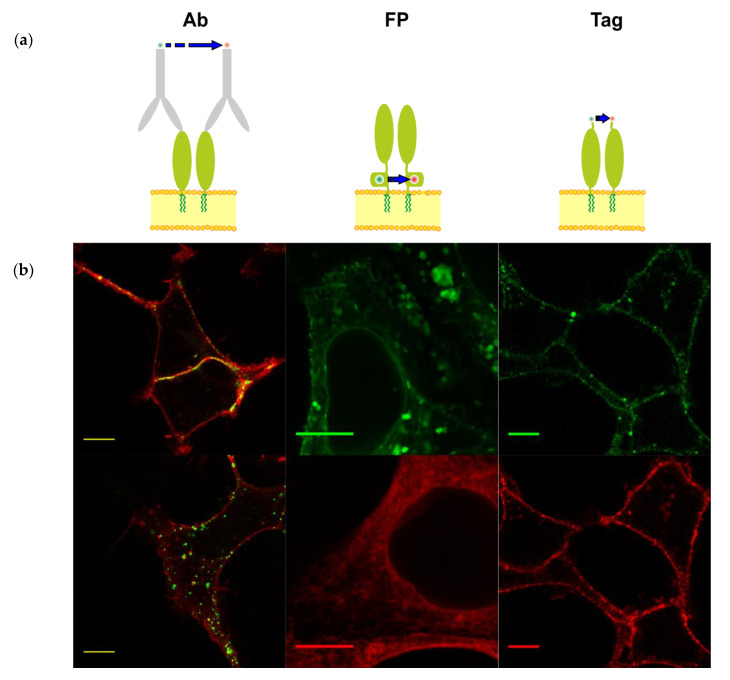
Schematic representation of the three methods for the fluorescent labeling of glycosylphosphatidylinositol-anchored proteins (GPI-APs) with two different fluorophores (donor and acceptor) for FRET experiments and the visualization of T-cadherin labeling in live cells. (**a**) GPI-APs are depicted as light green ovals and are anchored on the outer side of the plasma membrane (yellow). Blue arrows represent FRET. (Ab) Labeling antibodies conjugated with either donor (green) or acceptor (red) dyes (fluorescent protein (FP)). Labeling with donor (green) and acceptor (red) FPs fused into the juxtamembrane region of GPI-AP. (Tag). Peptide-tag based labeling within the GPI-AP with small synthetic dyes (donor in green and acceptor in red) by means of Sfp synthase. (**b**) The left panel shows representative confocal images of the distinct staining patterns of T-cadherin (green) after incubation with different antibodies against T-cadherin. Top—homogeneous membrane staining, R&D # BAF3264; bottom—punctated intracellular staining, ProSci # 3583. The plasma membrane was stained with membrane marker dye (red). The central panel shows representative confocal images of the different intracellular patterns of T-cad-TFP (green, vesicle and membrane staining) and T-cad-YFP (red, endoplasmic reticulum and membrane staining). The right panel shows representative confocal images of T-cadherin labeled with donor (green) and acceptor (red) dyes in the membrane. Scale bar—10 um.

We compared three different approaches to labeling GPI-APs—using fluorescent antibodies, fluorescent proteins, and a small synthetic fluorescent dye (Figure 3a). We showed significant differences between these three approaches for GPI-AP T-cadherin (Figure 3) (for more details, see [20]). The labeling of T-cadherin with a specific primary and fluorescent-dye-conjugated secondary antibody in live cells while using recommended amounts and incubation times for staining induced artificial clustering and internalization (Figure 3b). A similar experiment with a biotinylated primary antibody and fluorescent streptavidin allowed for us to retain a portion of T-cadherin on the plasma membrane. Different antibodies produced distinct staining patterns, punctate or homogeneous (Figure 3b, “Ab”), depending on the antibody properties (likely specificity and cross-reactivity).

The creation of a genetically coded chimera of the POI and a FP is another popular approach for fluorescent labeling. We inserted TFP as a donor and YFP as an acceptor in the same site of T-cadherin. However, the effect of the inserts of TFP and YFP was different (Figure 3b, “FP”). T-cadherin-TFP was localized on the plasma membrane and in some intracellular vesicles, whereas T-cadherin-YFP was mostly localized in the endoplasmic reticulum (Figure 3b, [20]). T-cadherin labeling with FPs affected intracellular localization, depending on maturation and folding, as we demonstrated using structure modeling, Western blotting, colocalization analysis of TFP and YFP, and a comparison of localization of FP and specific markers of the plasma membrane, endoplasmic reticulum [20]. The insertion of YFP distorted the structure and localization of T-cadherin, and we showed the low colocalization YFP and TFP making this strategy unfeasible for the analysis of T-cadherin surface distribution. In the work by Tyrberg and colleagues, the FP-fused T-cadherin chimera with mRFP was localized mostly inside cells and, possibly, was also misfolded [44]. Our result showed that not only the insertion site but also the nature of FP may affect POI properties and, in our case, it was better to find another approach.

We inserted the short peptide tag S6 [23] into T-cadherin (for the criterion for a suitable insertion site, see Section 3.1) and labeled the tagged protein using the enzyme phosphopantetheinyl transferase, namely Sfp-synthase, which attaches derivatives of coenzyme A to S6, to minimize structural distortion and probe T-cadherin clustering. We successfully labeled T-cadherin with a synthetic probe via the enzyme on the cell’s surface (Figure 3, “Tag”). This labeling preserves the localization and function of T-cadherin (Section 3.2, [20]).

The small-tag labeling approach has major advantages (Table 1). It minimizes the influence of fluorescent labeling on the protein’s properties, since the tag size (S6 peptide contains 12 amino acid residues) and fluorophores are much smaller than the fluorescent proteins (2.5 kDa vs. 27 kDa). One can restrict labeling to the exposed proteins in living cells using a cell-impermeable fluorescent substrate. With one genetic construct or transduced cell line, one can use a broad spectrum of synthetic dyes to label the target protein. Synthetic fluorescent dyes are not prone to dimerization, unlike FPs. Moreover, they offer superior control of the donor-to-acceptor ratio can be easily achieved at the labeling step. Small synthetic fluorophores are usually brighter and more stable than FPs and, in some cases, are indispensable, especially for super-resolution techniques. More precise information about the distance between POIs can be obtained by FRET, because FRET efficiency depends not only on distance, but also on the dipole orientation factor ($\kappa^{2}$) due to the small size, weight, and rotational time of organic fluorophores. The sizes of the FPs (2–4 nm) are comparable with the Förster radii *R*_0_ and could significantly affect the calculated distance between the POIs from the FRET experiment. The rotational correlation times of the FRs are in the range of 15–20 ns, which exceeds the fluorescence lifetime (in the range of 2–4 ns) [45]. This makes the estimation of the $\kappa^{2}$ factor for FPs highly uncertain. Synthetic fluorophores are smaller, and their rotation diffusion is quite fast [46]. The rotational correlation time and fluorescent lifetime of bound cyanine are comparable in aqueous solutions (0.2–4 ns) [47], allowing for the dipole orientation factor $\kappa^{2}$ for FRET to be averaged and making that value very likely to be close to the average value of ⅔. This consideration is valid in our case due to the rotational freedom provided by coenzyme A, which links fluorophore to the peptide tag.

All three methods have their own disadvantages and advantages and, in each particular case, the optimization of fluorescent labeling is necessary. Staining with fluorophore-conjugated antibodies is useful. For example, a study of GPI-anchored 5’-nucleotidase distribution on the plasma membrane using FRET imaging followed labeling with fluorophore-conjugated monovalent Fab and the bivalent IgG antibody [8]. There are examples of the successful generation of functional fusions of GPI-AP with FP that can be used for FRET studies too [21,22].

### 3.2. Validation of Chimeric Proteins with S6-Tag

**Figure 4:**
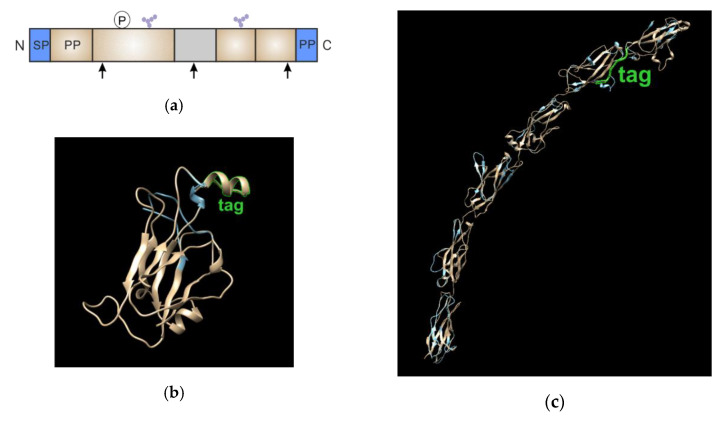
Insertion of the labeling tag to the GPI-anchored receptors. (**a**). Schematic drawing of the general GPI-anchored receptor. Arrows—potential sites of tag insertion, SP—signal peptide, PP— prodomain (cleaved out during processing), P—phosphorylation site; glycosylation sites are indicated as violet hexagons representing glycan chain, and the non-structured fragment is shown in grey; (**b**). The three-dimensional (3D) structure of ephrin-A1 with (beige) and without (blue) tag; the tag S6 is shown in green (**c**). The 3D structure of T-cadherin with (beige) and without (blue) tag; the tag S6 is shown in green.

T-cadherin-S6 and ephrin-A1-S6 were constructed by inserting the sequence encoding the S6-peptide [23]. We followed several criteria to preserve the three-dimensional (3D) structure of the labeled proteins and achieve successful labeling for the FRET experiments to select the tag insertion site (Figure 4a):

1) the inserted tag should be present in the mature protein;
2) the introduced peptide or polypeptide should not interfere with the enzymes involved in the post-translational modification of the POI (attachment of the GPI anchor, glycosylation, proteolysis, etc.);
3) modification should not affect the structure of the extracellular domains and ligand-binding sites; and,
4) the label should be positioned as close as possible to the part of the protein participating in dimerization to maximize the FRET signal.

We compared the structures of wild-type (WT) GPI-APs, T-cadherin-S6, and ephrin-A1-S6 (Figure 4b,c), and did not note any significant differences.

**Figure 5:**
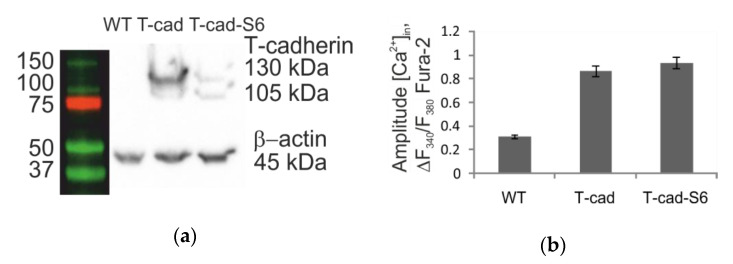

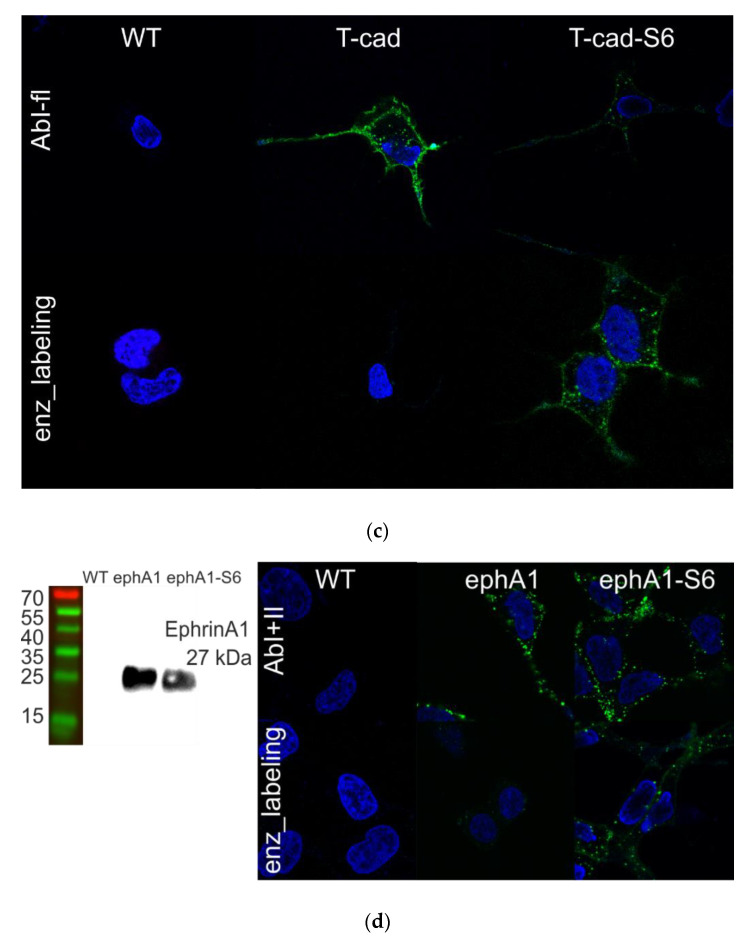
Comparative analysis of wild-type (WT) cells, cells expressing T-cadherin/ephrin A1, and cells expressing T-cad-S6/ephrin-A1-S6. (**a**) The immunoblots of the lysates of three cell types: WT, T-cad, and T-cad-S6. The molecular weights of the T-cadherin and T-cadherin-S6 are very similar: 105 kDa - mature protein and 130 kDa - protein with prodomain. Beta-actin was used as the loading control (**b**) The amplitudes of the LDL-induced [Ca^2+^]_i_ responses of the three cell types: WT, T-cad, and T-cad-S6. The amplitudes of the cells expressing T-cadherin and cells expressing T-cad-S6 are the same (for more details, see [20]). (**c**) Staining of T-cadherin with APC-labeled antibodies (AbI-fl) or the selective enzyme-mediated labeling of WT, T-cad, and T-cad-S6 cells with small dyes. (**d**) Left ─ Immunoblots of lysates obtained from the three cell types: WT, ephrin-A1, and ephrin A-S6. The molecular weights of ephrin-A1 and ephrinA1-S6 are apparently equal. The equal amount of total protein we used in each line. Right—Antibody staining of ephrin A1 with primary and secondary antibodies (AbI+II) or the selective enzyme-mediated labeling of WT, ephrin-A1, and ephrin-A1-S6 cells.

The chimeras were transfected into the HEK293 cell line, as these cells do not express WT T-cadherin and ephrin-A1. Intracellular localization and surface expression of T-cadherin and ephrin-A1 were essentially unaffected by the insertion of the tag, as shown by antibody staining followed by the confocal fluorescence microscopy (Figure 5) and flow cytometry analysis (data not shown). Untransfected wild type cells were used in order to confirm the specificity of antibody staining (Figure 5 WT).

The functionality of recombinant T-cadherin was confirmed by its ability to mediate calcium signaling upon binding with LDL [20]. We recorded a comparable calcium response to LDL in cells with unmodified and S6-tagged T-cadherin (Figure 5b).

Thus, the chimeras were correctly GPI anchored, sorted to the cell surface, and had biological activities.

### 3.3. FRET between Labeled Molecules on the Cell Surface

As mentioned above, GPI-APs may be clustered on the cell surface without ligands; their distribution depends on the GPI anchor’s composition and the structure of the ectodomain. Our labeling approach using two different dyes allowed for us to obtain information regarding the distribution of GPI-APs in different ways.

**Figure 6:**
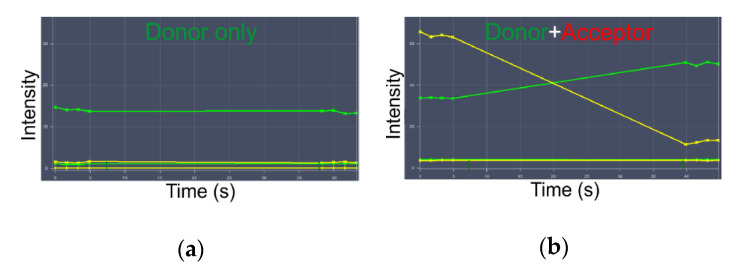
Acceptor photobleaching for the analysis of FRET efficiency in unstimulated cells. Green lines—fluorescence in the donor channel; yellow lines—fluorescence in the acceptor channel. (**a**) Sample with donor only; fluorescence in the control (background) and object (cell labeled donor) Regions of Interest (ROIs). (**b**) Samples with donors and acceptors; fluorescence in the control (background) and object (cell labeled donor and acceptor) ROIs.

First, we used the FRET detected by acceptor photobleaching using confocal microscopy. Acceptor photobleaching is a useful and simple method because all of the parameters needed for calculation can be obtained from the same image. However, the experimental conditions must be carefully chosen. This method requires a photostable donor that is not detectably photobleached during measurement and a photolabile acceptor, which can be completely photobleached in a relatively short time [35]. Before engaging in acceptor photobleaching measurements, we checked the photobleaching donor in the samples with donors only, and different times were required for completely bleaching the acceptor (Figure 6). We detected a reliable FRET signal in the cells with T-cadherin (Figure 6). Using this method, it was found that FRET efficacy is 11.8 ± 1.8% on the surface of living cells in a medium without ligands, according to Equation (3). It is preferable to perform this type of FRET determination on fixed cells since the proteins in live cells are mobile, and acceptor photobleaching is a relatively slow process.

It should be noted that, with confocal fluorescence microscopy and total internal reflection fluorescence (TIRF) microscopy, it is possible to focus on the basal cell membrane (a part of the plasma membrane that is in contact with the coverglass) and filter out-of-focus fluorescence to increase the signal-to-noise ratio for the detection of membrane-localized fluorescence and the FRET signal. Membrane-specific labeling is much more effective in localizing fluorescence on the plasma membrane than confocal optical filtering and it provides the unique possibility to restrict fluorescence to the plasma membrane and detect it by flow cytometry. Some scientists call the FRET measured by flow cytometry FACS-FRET (fluorescence-activated cell sorting-FRET) or FCET (flow cytometric FRET) [42,48,49]. When compared to microscopy, this method has several advantages: (1) it is intrinsically quantitative and has a much wider dynamic range of fluorescence detection with a linear response curve; (2) the detected signal is averaged for the entire cell surface; and, (3) it analyzes thousands of cells within seconds, making it suitable for kinetic measurements and increasing the reliability of the data. As a downside, flow cytometry does not provide subcellular distribution information and does not allow for us to measure the kinetics of a certain cell, but only for a whole population. Another disadvantage is that flow cytometry is used to analyze cells in a suspension, and the spatiotemporal organization of membrane proteins might be different in adherent and suspension cells. A slight dilution of the ligand during the flow cytometry procedure might also affect the measurement of the dissociation of the ligand-induced receptor dimer complex.

The FRET efficacy of ephrin-A1 was 14% and T-cadherin was less than 10% based on median fluorescent intensities and the equations described in [42,43]. Despite we detected FRET signal (using confocal microscopy or flow cytometry) of T-cadherin-S6, the FRET signal of T-cadherin-S6-S6 chimera was higher. It means that not all of the receptors are clusters and the ligand could induce clusterization.

### 3.4. Real-Time Kinetics of FRET

**Figure 7:**
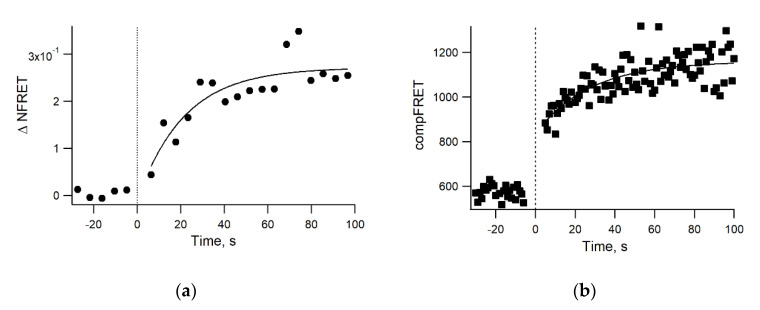
Representative kinetics of FRET (dimerization of T-cadherin) following the administration of 10 ug/mL adiponectin in HEK293 living cells. (**a**) The change in the normalized FRET compared to the baseline according to confocal laser scanning microscopy. Each point corresponds to the average NFRET value of a single microscopic image (a few thousand nonzero points). (**b**) The change in the compensated FRET signal measured by flow cytometry. Each point corresponds to the median fluorescence intensity in the 670/30 nm channel after 561 nm excitation of a few thousands living cells gated on the basis of forward and side light scattering. The solid lines represent the fitting of the data by exponential curves.

It is important to receive real-time information on GPI-AP cluster formation upon ligand administration for live cells. We showed an increase in the FRET signal after the addition of 10 μg/mL adiponectin with similar kinetics for both the microscopy and flow cytometry settings (Figure 7). The GPI-AP clusters were stable at the end of the timed measurement. The results of the flow cytometry and confocal microscopy analysis showed good agreement and allowed for us to obtain quantitative information about dimerization dynamics (characteristic exponential raise time in range 20–40 s) [20]. Moreover, these values are in agreement with independent data on the rate constants of adiponectin binding to T-cadherin in a cell-free system) [20,50].

## 4. Conclusions

Since its discovery, FP technology has become a conventional and widely used tool for the analysis of protein dynamics and interactions in living cells under various techniques, including FRET [51]. However, FPs have several drawbacks, including a tendency to dimerization and distort the target protein’s properties [11,52]. This issue is particularly problematic for GPI-APs and small proteins, such as ephrin-A. Here, we showed how crucial FP insertion can be for the properties of T-cadherin and compared fluorescent labeling of GPI-anchored T-cadherin with antibodies, fluorescent proteins, and enzymatically modified small peptide tags. We chose tag mediated enzymatic labeling with Sfp synthase, which offers nearly the best balance between protein distortion, selectivity, and versatility among the various suitable techniques, to minimize the deleterious effects of labeling [53]. However, this technique has not been evaluated for the analysis of FRET in living cells. In this paper, we considered the practical aspects of GPI-AP labeling and developed an analysis of their clustering on the plasma membrane via sensitized acceptor emissions—a very affordable method for FRET detection. We demonstrated several advantages of the proposed technique: the use of internal peptide tags to label GPI-APs (by the example of T-cadherin and ephrin-A1); the use of specific membrane labeling to improve the signal to noise ratio and facilitate data processing; and, the ability to use both confocal microscopy and flow cytometry to measure protein clustering dynamics by FRET. In our companion paper, we used this approach to study the T-cadherin rearrangements that are responsible for distinct responses to different ligands [20]. We believe that this approach is worth considering for the study of dynamic interactions in plasma membranes and our efforts will aid future research into membrane receptors.

## Figures and Tables

**Table 1:** Side by side comparison of the three methods of membrane receptor labeling for heteroFRET measurements.

	With Fluorescent Antibodies	Chimeras with Fluorescent Proteins	Enzyme-Mediated Fluorescence Labeling of Small Tags
Genetic modification of the protein of interest (POI)	No	Yes (two constructs)	Yes (one construct)
Site specific labeling of the POI	In some cases, the epitope is unknown	Yes	Yes
Possibility to use the same cells for control (only donor or acceptor) experiments	Yes	No	Yes
Selective labeling of surface-exposed proteins	Yes	No	Yes
Approximate volume of the fluorescent label	400 nm^3^	30 nm^3^	2 nm^3^
Approximate molecular weight of the label	150 kDa	27 kDa	2.5 kDa
Time for labeling reaction	15–120 min	0 min	15–60 min
Control of donor: acceptor ratio	Complicated	Complicated	Yes
Artificial dimerization	In some cases, the antibodies stimulate dimerization	In some cases, the FPs form a dimer	Not detected

## References

[1] Bocharov E.V., Sharonov G.V., Bocharova O.V., Pavlov K.V. (2017). Conformational transitions and interactions underlying the function of membrane embedded receptor protein kinases. Biochim. Biophys. Acta Biomembr..

[2] Sharma P., Varma R., Sarasij R.C., Gousset K., Krishnamoorthy G., Rao M., Mayor S. (2004). Nanoscale Organization of Multiple GPI-Anchored Proteins in Living Cell Membranes. Cell.

[3] Suzuki K.G., Kasai R.S., Hirosawa K.M., Nemoto Y.L., Ishibashi M., Miwa Y., Fujiwara T.K., Kusumi A. (2012). Transient GPI-anchored protein homodimers are units for raft organization and function. Nat. Chem. Biol..

[4] Suzuki K.G., Fujiwara T.K., Sanematsu F., Iino R., Edidin M., Kusumi A. (2007). GPI-anchored receptor clusters transiently recruit Lyn and G alpha for temporary cluster immobilization and Lyn activation: Single-molecule tracking study 1. J. Cell Biol..

[5] Sevcsik E., Brameshuber M., Folser M., Weghuber J., Honigmann A., Schutz G.J. (2015). GPI-anchored proteins do not reside in ordered domains in the live cell plasma membrane. Nat. Commun..

[6] Lebreton S., Zurzolo C., Paladino S. (2018). Organization of GPI-anchored proteins at the cell surface and its physiopathological relevance. Crit. Rev. Biochem. Mol. Biol..

[7] Kusumi A., Fujiwara T.K., Morone N., Yoshida K.J., Chadda R., Xie M., Kasai R.S., Suzuki K.G. (2012). Membrane mechanisms for signal transduction: The coupling of the meso-scale raft domains to membrane-skeleton-induced compartments and dynamic protein complexes. Semin. Cell Dev. Biol..

[8] Kenworthy A.K., Edidin M. (1998). Distribution of a glycosylphosphatidylinositol-anchored protein at the apical surface of MDCK cells examined at a resolution of <100 A using imaging fluorescence resonance energy transfer. J. Cell Biol..

[9] Stryer L. (1978). Fluorescence energy transfer as a spectroscopic ruler. Annu. Rev. Biochem..

[10] Rao M., Mayor S. (2005). Use of Forster’s resonance energy transfer microscopy to study lipid rafts. Biochim. Biophys. Acta.

[11] Piston D.W., Kremers G.J. (2007). Fluorescent protein FRET: The good, the bad and the ugly. Trends Biochem. Sci..

[12] Sun Y., Wallrabe H., Seo S.A., Periasamy A. (2011). FRET microscopy in 2010: The legacy of Theodor Forster on the 100th anniversary of his birth. Chemphyschem A Eur. J. Chem. Phys. Phys. Chem..

[13] Shrestha D., Jenei A., Nagy P., Vereb G., Szollosi J. (2015). Understanding FRET as a research tool for cellular studies. Int. J. Mol. Sci..

[14] Szollosi J., Vereb G., Nagy P. (2016). The flow of events: How the sequence of molecular interactions is seen by the latest, user-friendly high throughput flow cytometric FRET. Cytom. A.

[15] Denzel M.S., Scimia M.C., Zumstein P.M., Walsh K., Ruiz-Lozano P., Ranscht B. (2010). T-cadherin is critical for adiponectin-mediated cardioprotection in mice. J. Clin. Investig..

[16] Parker-Duffen J.L., Nakamura K., Silver M., Kikuchi R., Tigges U., Yoshida S., Denzel M.S., Ranscht B., Walsh K. (2013). T-cadherin Is Essential for Adiponectin-mediated Revascularization. J. Biol. Chem..

[17] Balatskaya M.N., Balatskii A.V., Sharonov G.V., Tkachuk V.A. (2016). T-cadherin as a novel receptor regulating metabolism in the blood vessel and heart cells: From structure to function. J. Evol. Biochem. Phys..

[18] Fujishima Y., Maeda N., Matsuda K., Masuda S., Mori T., Fukuda S., Sekimoto R., Yamaoka M., Obata Y., Kita S. (2017). Adiponectin association with T-cadherin protects against neointima proliferation and atherosclerosis. FASEB J..

[19] Obata Y., Kita S., Koyama Y., Fukuda S., Takeda H., Takahashi M., Fujishima Y., Nagao H., Masuda S., Tanaka Y. (2018). Adiponectin/T-cadherin system enhances exosome biogenesis and decreases cellular ceramides by exosomal release. JCI Insight.

[20] Balatskaya M.N., Sharonov G.V., Baglay A.I., Rubtsov Y.P., Tkachuk V.A. (2019). Different spatiotemporal organization of GPI-anchored T-cadherin in response to low-density lipoprotein and adiponectin. Biochim. Biophys. Acta Gen. Subj..

[21] Caiolfa V.R., Zamai M., Malengo G., Andolfo A., Madsen C.D., Sutin J., Digman M.A., Gratton E., Blasi F., Sidenius N. (2007). Monomer dimer dynamics and distribution of GPI-anchored uPAR are determined by cell surface protein assemblies. J. Cell Biol..

[22] Tavares E., Macedo J.A., Paulo P.M., Tavares C., Lopes C., Melo E.P. (2014). Live-cell FRET imaging reveals clustering of the prion protein at the cell surface induced by infectious prions. Biochim. Biophys. Acta.

[23] Zhou Z., Cironi P., Lin A.J., Xu Y., Hrvatin S., Golan D.E., Silver P.A., Walsh C.T., Yin J. (2007). Genetically encoded short peptide tags for orthogonal protein labeling by Sfp and AcpS phosphopantetheinyl transferases. ACS Chem. Biol..

[24] Yin J., Straight P.D., McLoughlin S.M., Zhou Z., Lin A.J., Golan D.E., Kelleher N.L., Kolter R., Walsh C.T. (2005). Genetically encoded short peptide tag for versatile protein labeling by Sfp phosphopantetheinyl transferase. Proc. Natl. Acad. Sci. USA.

[25] Kelley L.A., Mezulis S., Yates C.M., Wass M.N., Sternberg M.J. (2015). The Phyre2 web portal for protein modeling, prediction and analysis. Nat. Protoc..

[26] Pettersen E.F., Goddard T.D., Huang C.C., Couch G.S., Greenblatt D.M., Meng E.C., Ferrin T.E. (2004). UCSF Chimera—A visualization system for exploratory research and analysis. J. Comput. Chem..

[27] Ciatto C., Bahna F., Zampieri N., VanSteenhouse H.C., Katsamba P.S., Ahlsen G., Harrison O.J., Brasch J., Jin X., Posy S. (2010). T-cadherin structures reveal a novel adhesive binding mechanism. Nat. Struct. Mol. Biol..

[28] Nekrasova O.V., Sharonov G.V., Tikhonov R.V., Kolosov P.M., Astapova M.V., Yakimov S.A., Tagvey A.I., Korchagina A.A., Bocharova O.V., Wulfson A.N. (2012). Receptor-binding domain of ephrin-A1: Production in bacterial expression system and activity. Biochemistry.

[29] Himanen J.P., Goldgur Y., Miao H., Myshkin E., Guo H., Buck M., Nguyen M., Rajashankar K.R., Wang B., Nikolov D.B. (2009). Ligand recognition by A-class Eph receptors: Crystal structures of the EphA2 ligand-binding domain and the EphA2/ephrin-A1 complex. EMBO Rep..

[30] Inoue H., Nojima H., Okayama H. (1990). High efficiency transformation of Escherichia coli with plasmids. Gene.

[31] Yin J., Lin A.J., Golan D.E., Walsh C.T. (2006). Site-specific protein labeling by Sfp phosphopantetheinyl transferase. Nat. Protoc..

[32] Zhang A., Sun L., Buswell J., Considine N., Ghosh I., Masharina A., Noren C., Xu M.Q. (2011). Fluorescent site-specific labeling of Escherichia coli expressed proteins with Sfp phosphopantetheinyl transferase. Methods Mol. Biol..

[33] Lakowicz J.R. (1999). Principles of Fluorescence Spectroscopy.

[34] Shapiro L., Fannon A.M., Kwong P.D., Thompson A., Lehmann M.S., Grubel G., Legrand J.F., Als-Nielsen J., Colman D.R., Hendrickson W.A. (1995). Structural basis of cell-cell adhesion by cadherins. Nature.

[35] Berney C., Danuser G. (2003). FRET or no FRET: A quantitative comparison. Biophys. J..

[36] Feige J.N., Sage D., Wahli W., Desvergne B., Gelman L. (2005). PixFRET, an ImageJ plug-in for FRET calculation that can accommodate variations in spectral bleed-throughs. Microsc. Res. Tech..

[37] Sharonov G.V., Mozgovaja M.N., Astapova M.V., Kolosov P.M., Arseniev A.S., Feofanov A.V., Méndez-Vilas A. (2012). Study of EphA2 dimerization and clusterization in living cells using sensitized acceptor emission in FRET pair. Current Microscopy Contributions to Advances in Science and Technology.

[38] Xia Z., Liu Y. (2001). Reliable and global measurement of fluorescence resonance energy transfer using fluorescence microscopes. Biophys. J..

[39] Gordon G.W., Berry G., Liang X.H., Levine B., Herman B. (1998). Quantitative fluorescence resonance energy transfer measurements using fluorescence microscopy. Biophys. J..

[40] Roederer M. (2002). Compensation in flow cytometry. Curr. Protoc. Cytom..

[41] Tron L., Szollosi J., Damjanovich S., Helliwell S.H., Arndt-Jovin D.J., Jovin T.M. (1984). Flow cytometric measurement of fluorescence resonance energy transfer on cell surfaces. Quantitative evaluation of the transfer efficiency on a cell-by-cell basis. Biophys. J..

[42] Ujlaky-Nagy L., Nagy P., Szollosi J., Vereb G. (2018). Flow Cytometric FRET Analysis of Protein Interactions. Methods Mol. Biol..

[43] Szentesi G., Horváth G., Bori I., Vámosi G., Szöllősi J., Gáspár R., Damjanovich S., Jenei A., Mátyus L. (2004). Computer program for determining fluorescence resonance energy transfer efficiency from flow cytometric data on a cell-by-cell basis. Comput. Methods Programs Biomed..

[44] Tyrberg B., Miles P., Azizian K.T., Denzel M.S., Nieves M.L., Monosov E.Z., Levine F., Ranscht B. (2011). T-cadherin (Cdh13) in association with pancreatic beta-cell granules contributes to second phase insulin secretion. Islets.

[45] Vogel S.S., Nguyen T.A., van der Meer B.W., Blank P.S. (2012). The impact of heterogeneity and dark acceptor states on FRET: Implications for using fluorescent protein donors and acceptors. PLoS ONE.

[46] Devauges V., Marquer C., Lecart S., Cossec J.C., Potier M.C., Fort E., Suhling K., Leveque-Fort S. (2012). Homodimerization of amyloid precursor protein at the plasma membrane: A homoFRET study by time-resolved fluorescence anisotropy imaging. PLoS ONE.

[47] Sanborn M.E., Connolly B.K., Gurunathan K., Levitus M. (2007). Fluorescence properties and photophysics of the sulfoindocyanine Cy3 linked covalently to DNA. J. Phys. Chem. B.

[48] Banning C., Votteler J., Hoffmann D., Koppensteiner H., Warmer M., Reimer R., Kirchhoff F., Schubert U., Hauber J., Schindler M. (2010). A flow cytometry-based FRET assay to identify and analyse protein-protein interactions in living cells. PLoS ONE.

[49] Szollosi J., Damjanovich S., Matyus L. (1998). Application of fluorescence resonance energy transfer in the clinical laboratory: Routine and research. Cytometry.

[50] Fukuda S., Kita S., Obata Y., Fujishima Y., Nagao H., Masuda S., Tanaka Y., Nishizawa H., Funahashi T., Takagi J. (2017). The unique prodomain of T-cadherin plays a key role in adiponectin binding with the essential extracellular cadherin repeats 1 and 2. J. Biol. Chem..

[51] Chudakov D.M., Matz M.V., Lukyanov S., Lukyanov K.A. (2010). Fluorescent proteins and their applications in imaging living cells and tissues. Physiol. Rev..

[52] Crivat G., Taraska J.W. (2012). Imaging proteins inside cells with fluorescent tags. Trends Biotechnol..

[53] Lotze J., Reinhardt U., Seitz O., Beck-Sickinger A.G. (2016). Peptide-tags for site-specific protein labelling in vitro and in vivo. Mol. Biosyst..

